# Trimebutine, a small molecule mimetic agonist of adhesion molecule L1, contributes to functional recovery after spinal cord injury in mice

**DOI:** 10.1242/dmm.029801

**Published:** 2017-09-01

**Authors:** Junping Xu, Chengliang Hu, Qiong Jiang, Hongchao Pan, Huifan Shen, Melitta Schachner

**Affiliations:** 1 Center for Neuroscience, Shantou University Medical College, 22 Xin Ling Road, Shantou, Guangdong 515041, People's Republic of China; 2 Keck Center for Collaborative Neuroscience and Department of Cell Biology and Neuroscience, Rutgers University, 604 Allison Road, Piscataway, NJ 08854, USA

**Keywords:** L1CAM mimetics, Small organic compounds, Trimebutine, Honokiol, Spinal cord injury, Mouse

## Abstract

Curing spinal cord injury (SCI) in mammals is a daunting task because of the lack of permissive mechanisms and strong inhibitory responses at and around the lesion. The neural cell adhesion molecule L1CAM (L1) has been shown to favor axonal regrowth and enhance neuronal survival and synaptic plasticity but delivery of full-length L1 or its extracellular domain could encounter difficulties in translation to therapy in humans. We have, therefore, identified several small organic compounds that bind to L1 and stimulate neuronal survival, neuronal migration and neurite outgrowth in an L1-dependent manner. Here, we assessed the functions of two L1 mimetics, trimebutine and honokiol, in regeneration following SCI in young adult mice. Using the Basso Mouse Scale (BMS) score, we found that ground locomotion in trimebutine-treated mice recovered better than honokiol-treated or vehicle-receiving mice. Enhanced hindlimb locomotor functions in the trimebutine group were observed at 6 weeks after SCI. Immunohistology of the spinal cords rostral and caudal to the lesion site showed reduced areas and intensities of glial fibrillary acidic protein immunoreactivity in both trimebutine and honokiol groups, whereas increased regrowth of axons was observed only in the trimebutine-treated group. Both L1- and L1 mimetic-mediated intracellular signaling cascades in the spinal cord lesion sites were activated by trimebutine and honokiol, with trimebutine being more effective than honokiol. These observations suggest that trimebutine and, to a lesser extent under the present experimental conditions, honokiol have a potential for therapy in regeneration of mammalian spinal cord injuries.

## Abstract (precis)

**Summary:** Small molecule L1CAM mimetics, showing L1 functions by promoting neurite outgrowth and neuronal survival *in vitro*, are now shown *in vivo*, with correlated signal transduction, to promote recovery after injury.

## INTRODUCTION

Spinal cord injury (SCI) is a devastating neurological disease that can lead to life-long disability (Schwab et al., 2006; Silver and Miller, 2004). Treatments to facilitate recovery from injury have therefore been pursued intensely by many laboratories (Bracken, 1991; Bracken and Holford, 1993; Bracken et al., 1990, 1992; Hall, 1992; Hall and Springer, 2004). The poor efficiency of regeneration after SCI has been attributed to both the presence of inhibitory molecules at the site of injury and the low intrinsic capacity for axon growth in the adult mammalian central nervous system (CNS) (Liu et al., 2011). It is thus important to find effective molecules to promote axonal regeneration.

The neural cell adhesion molecule L1 (L1CAM) has been shown to enhance regeneration in the injured spinal cord and poses a promising therapeutic target (Chen et al., 2016; Jakovcevski et al., 2013; Loers et al., 2014a; Lutz et al., 2016; Yoo et al., 2014). L1 is a member of the immunoglobulin superfamily (Rathjen et al., 1987) and contains six immunoglobulin-like domains and five fibronectin type III domains, followed by a transmembrane domain and a short cytoplasmic domain. Homophilic and heterophilic interactions of L1 on the cell surface participate in nervous system development (Maness and Schachner, 2007; Zhang et al., 2008). L1 promotes axonal growth and myelination (Barbin et al., 2004; Mohajeri et al., 1996), prevents neuronal apoptosis (Chen et al., 1999) and enhances neuronal survival, axonal targeting, axonal regrowth/sprouting after injury and synaptic plasticity (Schäfer and Frotscher, 2012; Zhang et al., 2008). L1 receptor activation is regulated by calcium-dependent intracellular messengers in growth cones (Klinz et al., 1995). Within a cellular and molecular context of neuronal outgrowth, it is well accepted that the beneficial functions of L1 are exerted by signal transduction through the cognate Src-MAPK, PI3K/AKT and casein kinase 2 (CK2) pathways (Maness and Schachner, 2007; Poplawski et al., 2012; Wang and Schachner, 2015), as well as regulation by tyrosine phosphorylation (Garver et al., 1997). A prior study has demonstrated that L1 conjugated to an Fc fragment (L1-Fc) facilitates locomotor recovery in rats after SCI (Roonprapunt et al., 2003), and L1-transfected embryonic stem cells enhance survival and support regrowth of corticospinal tract axons in mice post-SCI (Chen et al., 2005). Adeno-associated virus-mediated L1 expression promotes functional recovery after SCI and reduces expression of the astrogliosis-driven increase of glial fibrillary acidic protein (GFAP) expression (Chen et al., 2007). However, application of L1-Fc, injection of stem cells overexpressing L1 and viral delivery of L1 is difficult in humans. Small organic molecules that have been approved by the US Food and Drug Administration are feasible in clinical trials because of well-defined toxicological and pharmacokinetic profiles, and ease of production. To identify L1 agonists with translational potential, a library of chemically synthesized small molecules was screened for L1 agonism, resulting in the identification of duloxetine, phenelzine sulfate, tacrine, ethinyl estradiol, crotamiton, honokiol, trimebutine maleate (trimebutine) and piceid (Kataria et al., 2016). *In vitro*, all identified L1-binding compounds stimulate neurite outgrowth, neuronal survival and migration, and enhance Schwann cell migration, proliferation and myelination of neurons. Furthermore, treatment with L1 agonists stimulate L1 expression and proteolysis and Erk activation in cultured cerebellar granule cells. In an *in vivo* study, duloxetine and piceid have been demonstrated to confer enhanced regeneration, correlating with enhanced survival, outgrowth and remyelination of motoneurons, and attenuated astrocyte and microglia activation in a mouse model of SCI (Kataria et al., 2016).

Based on these data, we tested another two small molecule mimetics of L1, trimebutine and honokiol, in an adult mouse SCI model, in the hope of finding more effective compounds for L1-mediated therapy applicable in patients. In the current study, we observed enhanced recovery of locomotion in SCI-lesioned mice treated with trimebutine, compared with mice treated with vehicle control. Moreover, increased intensity of βIII-tubulin and neurofilament 200 immunostaining, and reduced area, intensity and density of GFAP immunostaining of astrocytes plus the activation of L1-mediated signaling pathways, including MAPK, PI3K/AKT and CK2, indicate that trimebutine has potential for treating nervous system injuries in mammals.

## RESULTS

### Trimebutine promotes locomotion recovery after SCI

**Fig. 1.**
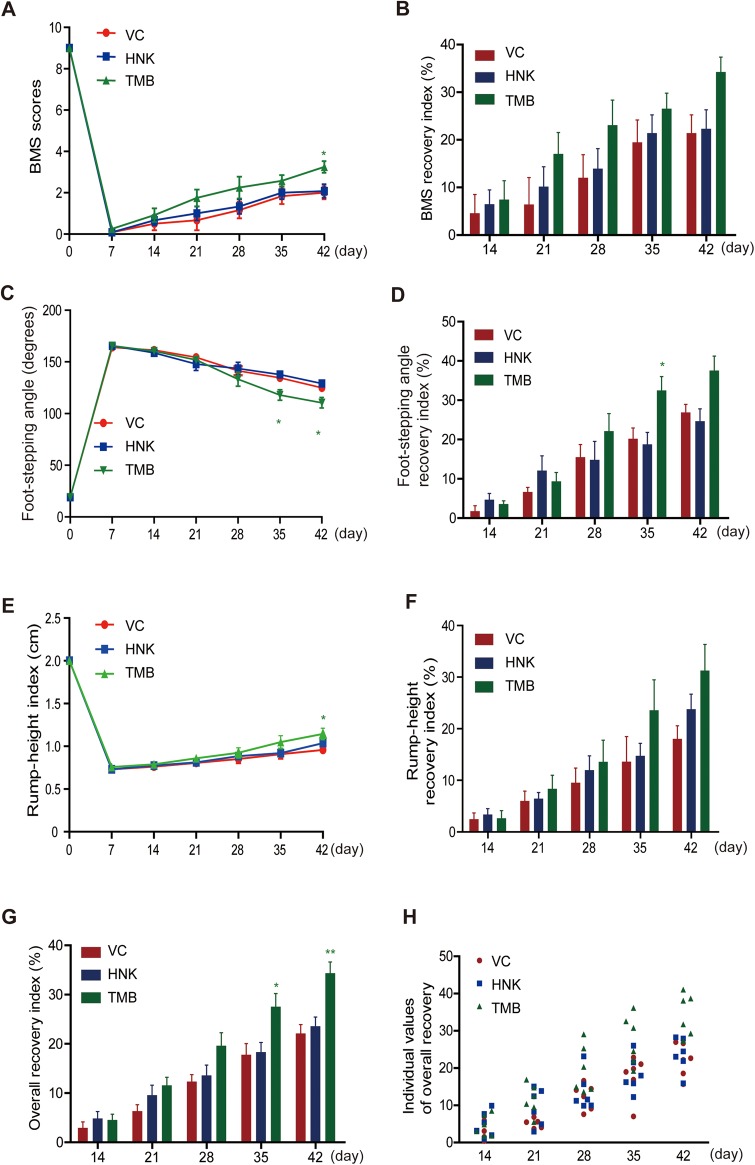
**Effects of trimebutine or honokiol on functional recovery from spinal cord injury.** (A-H) Time course and degree of recovery of locomotor functions after SCI in mice treated with vehicle control (VC), trimebutine (TMB) or honokiol (HNK). Shown are mean±s.e.m. values for BMS scores (A), foot-stepping angles (C), rump-height indices (E), and recovery indices (RI) after injury as measured by BMS scores (B), foot-stepping angles (D), and rump-height (F) at 1, 2, 3, 4, 5 and 6 weeks after SCI. Group mean values and individual values of overall recovery indices are shown in G and H, respectively. *P<0.05, **P<0.01 versus the vehicle control; one-way ANOVA with Tukey's post hoc test; *n*=6 mice/group.

Trimebutine and honokiol were tested in a mouse model of SCI involving severe locomotor paralysis, and recovery was assessed using the Basso Mouse Scale (BMS) score (Basso et al., 2006). BMS scores dramatically dropped from nine before SCI to zero 1 week after SCI in all groups (Fig. 1A), confirming the consistency and severity of the injury. From the second to sixth week after injury, better recovery was seen in mice treated with trimebutine, and this reached significance at 6 weeks (*P*=0.03 versus the vehicle control). Mice treated with honokiol showed slightly better recovery than vehicle control mice from 2 to 6 weeks after injury, as measured by BMS (Fig. 1B). Similarly, the values of foot-stepping angle and foot-stepping angle recovery index (RI) showed better recovery in mice treated with trimebutine (Fig. 1C,D), and the foot-stepping angle reached significance at 5 and 6 weeks (*P*=0.03 and *P*=0.048, respectively, versus the vehicle control) and foot-stepping angle RI at 5 weeks (*P*=0.036 versus the vehicle control), whereas there was no pronounced difference between honokiol-treated mice and vehicle control mice. There was an indication of a better recovery in the rump height index and the rump height RI in trimebutine-treated mice (Fig. 1E,F). Notably, the rump height index reached significance at 6 weeks (*P*=0.037 versus the vehicle control). Honokiol-treated mice showed slightly better recovery than vehicle control-treated mice by 6 weeks after SCI but this was not significant. Furthermore, analysis of the overall RI and the individual values of overall recovery (Fig. 1G,H) gave similar results, with the overall RI showing improvement in trimebutine-treated mice, reaching significance at 5 and 6 weeks (*P*=0.025 and *P*=0.002, respectively, versus the vehicle control). At 6 weeks, honokiol-treated mice showed no obvious difference from vehicle control-treated mice.

### Trimebutine and honokiol reduce lesion area and attenuate astrogliosis, and trimebutine increases βIII-tubulin neurofilament expression after SCI

**Fig. 2.**
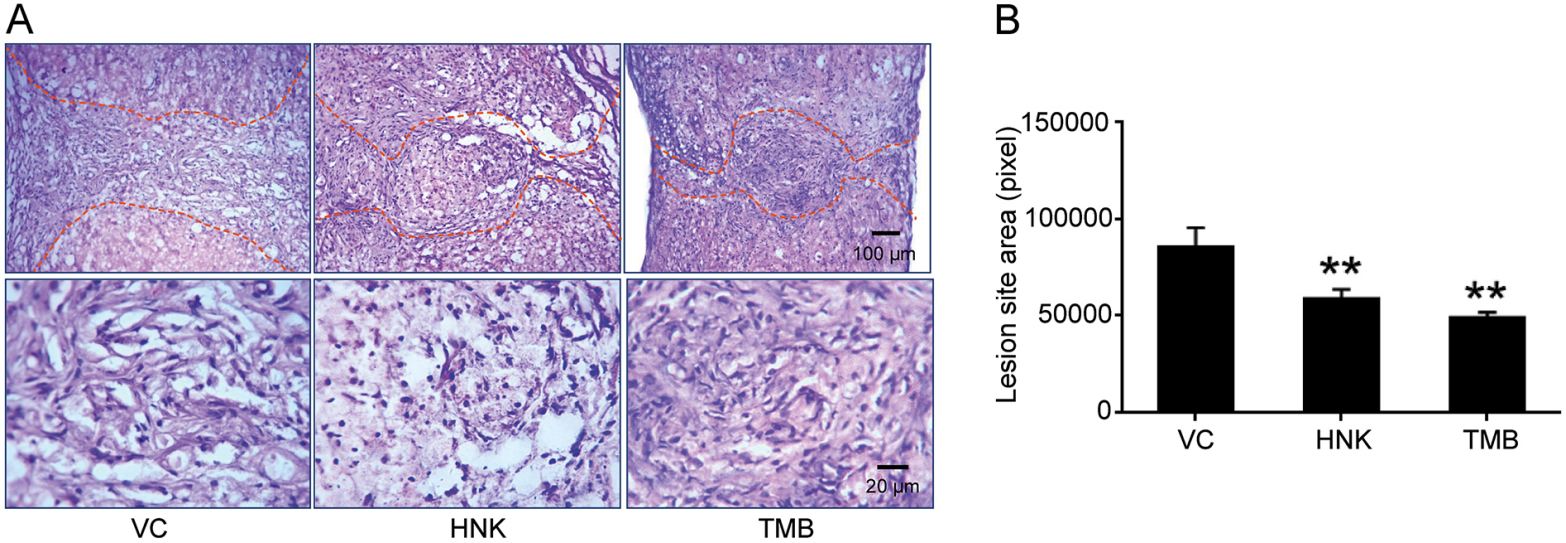
**Lesion areas at 6 weeks after spinal cord injury in mice treated with trimebutine or honokiol.** (A) Representative images of H&E-stained longitudinal sections at 6 weeks after SCI in vehicle control (VC), trimebutine (TMB) or honokiol (HNK) groups. (B) Quantitative analysis of lesion area at 6 weeks after SCI in vehicle control, trimebutine or honokiol groups. **P<0.01 versus the vehicle control; one-way ANOVA with Tukey's post hoc test; *n*=3 mice/group.

**Fig. 3.**
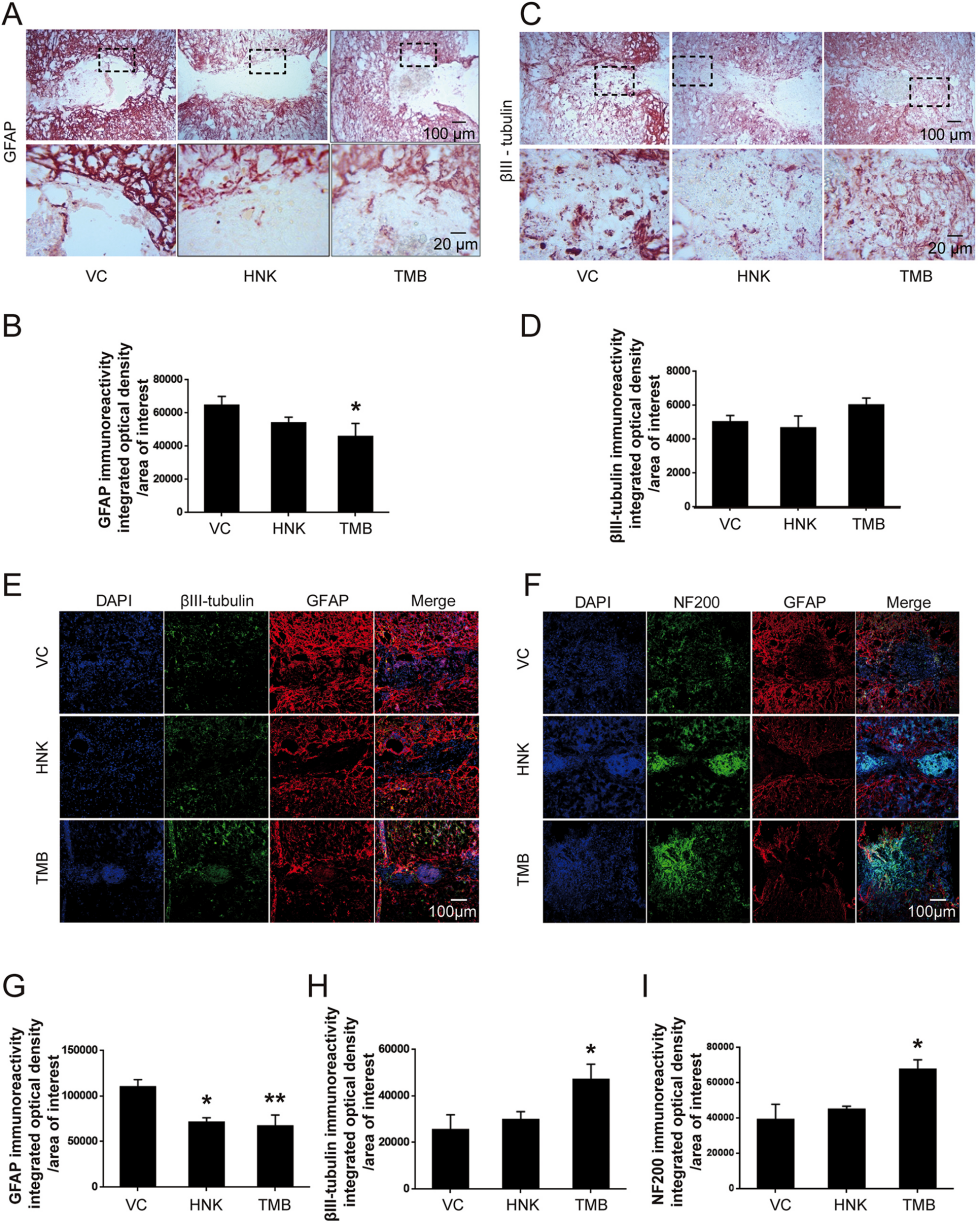
**GFAP, βIII-tubulin and NF200 immunoreactivity at 6 weeks after SCI in mice treated with trimebutine or honokiol.** (A-I) Representative micrographs of GFAP (A,E,F), βIII-tubulin (C,E) and NF200 (F) staining, and quantitative analysis of intensity of GFAP (B,G), βIII-tubulin (D,H) and NF200 (I) immunoreactivity. *P<0.05, **P<0.01 versus the vehicle control; one-way ANOVA with Tukey's post hoc test; *n*=3 mice/group. HNK, honokiol; TMB, trimebutine; VC, vehicle control.

The lesion areas of the injured spinal cords examined by Hematoxylin and Eosin (H&E) staining in longitudinal sections at 6 weeks were smaller in mice treated with either trimebutine or honokiol compared with mice treated with vehicle control (*P*=0.001 and *P*=0.01 for mice treated with trimebutine and honokiol, respectively, versus the vehicle control; Fig. 2A,B). Reactive astrocytes as measured by GFAP immunostaining within and in the vicinity of the lesion site form the glial scar tissue that is a molecular and mechanical barrier to axonal regrowth/sprouting (Liuzzi and Lasek, 1987). Immunostaining for GFAP (Fig. 3A,B,E-G) was less pronounced in mice treated with trimebutine or honokiol at 6 weeks, reaching statistically significance for trimebutine (*P*=0.032 in Fig. 3B and *P*=0.009 in 3G, versus vehicle control). Because trimebutine and honokiol have previously been shown to stimulate neurite outgrowth and neuronal survival *in vitro* (Kataria et al., 2016), neuronal markers such as βIII-tubulin and NF200 were examined. At 6 weeks after SCI, βIII-tubulin immunostaining indicated a higher neuronal density in trimebutine-treated mice at the lesion site (Fig. 3C,D,E,H; *P*=0.038 in 3H versus vehicle control). This trend was also observed with NF200 staining (Fig. 3F,I) and reached significance (*P*=0.021 versus vehicle control) in trimebutine-treated mice. Honokiol-treated mice tended to show the same differences in βIII-tubulin and NF200 immunostaining but these did not reach statistical significance.

### Trimebutine enhances protein levels of L1, pCK2α and mTOR, and reduces PTEN in the injured spinal cord

**Fig. 4.**
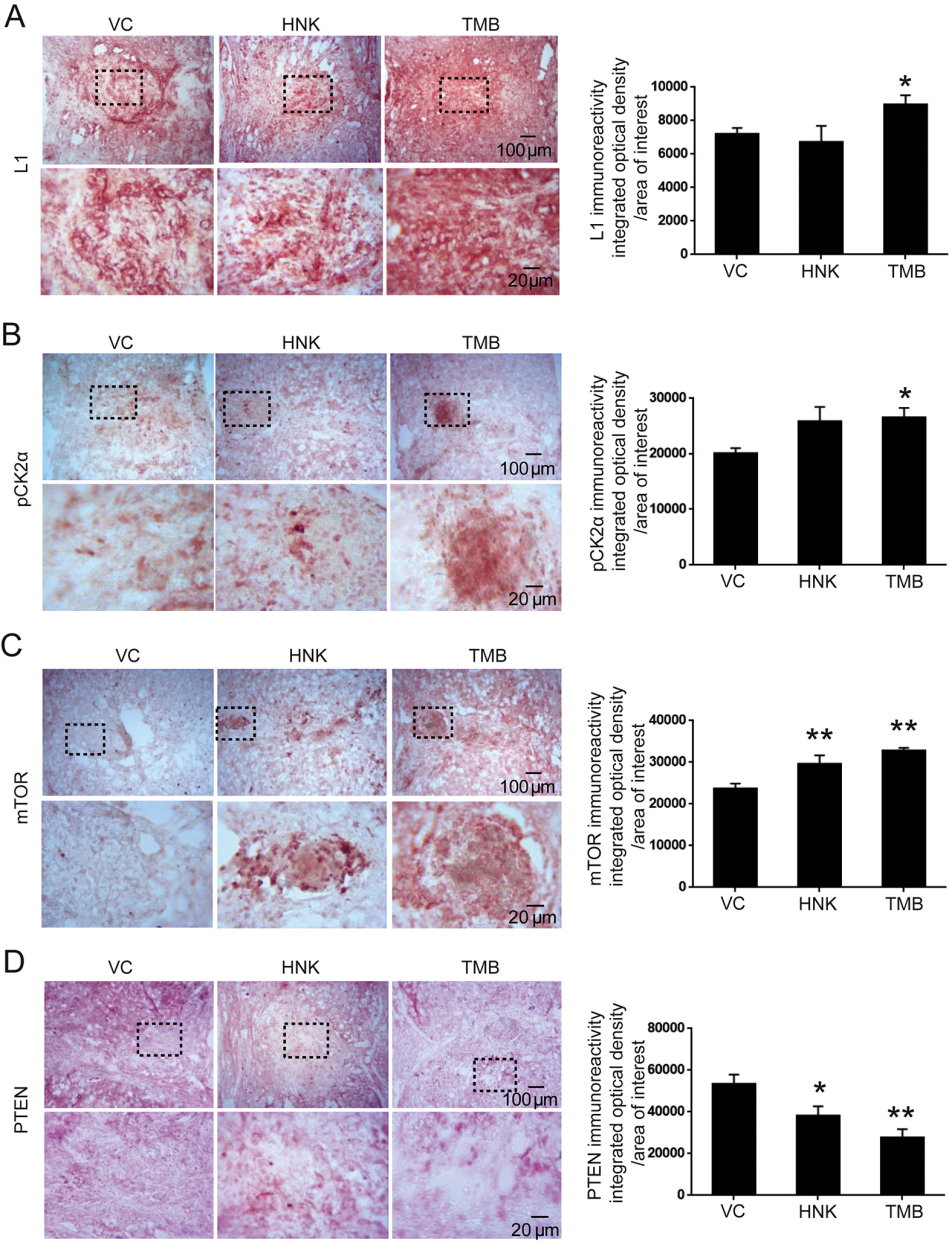
**L1, pCK2α, mTOR and PTEN immunoreactivity at 6 weeks after SCI in mice treated with trimebutine or honokiol.** (A-D) Representative images and quantitative analysis of L1 (A), pCK2α (B), mTOR (C) and PTEN (D) immunohistochemical staining. *P<0.05, **P<0.01 versus the vehicle control; one-way ANOVA with Tukey's post hoc test; *n*=3 mice/group. HNK, honokiol; TMB, trimebutine; VC, vehicle control.

In the L1-triggered and CK2-mediated signal transduction pathways, L1 increases pCK2α and mTOR, while reducing the level of PTEN *in vitro* (Wang and Schachner, 2015). We examined whether this pathway is also activated following SCI. At 6 weeks post-SCI, we measured protein expression by immunohistochemistry. In mice treated with trimebutine, we observed that levels of L1, pCK2α and mTOR were increased, whereas PTEN was decreased, with all changes achieving statistical significance compared with mice treated with vehicle control (*P*=0.042, *P*=0.021, *P*<0.00001 and *P*=0.001 for L1, pCK2α, mTOR and PTEN, respectively, versus vehicle control). Similar results were obtained in mice treated with honokiol, except that a change in L1 expression was not apparent (*P*=0.009 and *P*=0.022 for mTOR and PTEN, respectively, versus vehicle control; Fig. 4).

### Trimebutine increases L1- and pCK2α-immunopositive neurons in the lesion center of the injured spinal cord

**Fig. 5.**
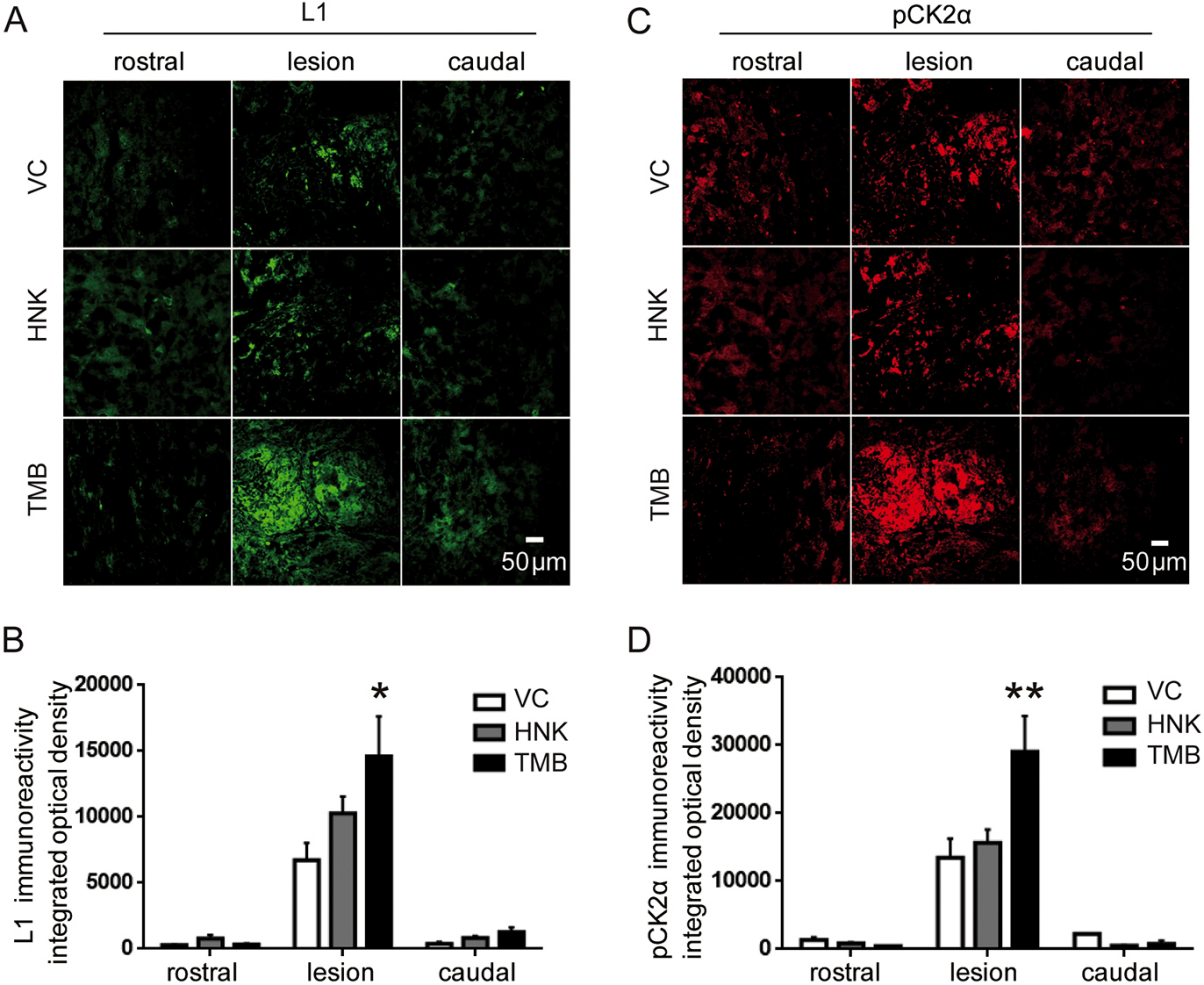
**Analysis of L1 and pCK2α immunofluorescence intensities rostral, central and caudal to the lesion site of the injured spinal cord at 6 weeks post-SCI in mice treated with trimebutine or honokiol.** (A,C) Representative images of L1 (A) and pCK2α (C) rostral (1 mm to the lesion center), central and caudal (1 mm to the lesion center) to the lesion site. (B,D) Quantitative analysis of L1 (B) and pCK2α (D) immunoreactivities. *P<0.05, **P<0.01 versus the vehicle control; one-way ANOVA with Tukey's post hoc test; *n*=3 mice/group. HNK, honokiol; TMB, trimebutine; VC, vehicle control.

L1 supports neurite outgrowth, neuronal adhesion, fasciculation, migration, axonal guidance and myelination, as well as synaptic plasticity. Similar to these functions of L1, CK2 promotes nerve growth factor-mediated neuronal survival (Lee et al., 2012). Therefore, using immunofluorescence staining of longitudinal sections, we assessed the staining intensity of L1- and pCK2α-immunopositive axons within the lesion site and rostral and caudal to the lesion (Fig. 5). At 6 weeks after injury, trimebutine- and honokiol-treated mice showed a higher staining intensity of L1 and pCK2α, compared with vehicle control-treated mice. Notably, changes observed in the trimebutine-treated mice achieved significance (*P*=0.014 and *P*=0.007 for L1 and pCK2α, respectively, versus vehicle control). No obvious difference was observed for L1 and pCK2α 1 mm rostral to the lesion and 1 mm caudal to lesion.

### Trimebutine triggers L1-mediated intracellular signaling cascades in the lesion site of the injured spinal cord

**Fig. 6.**
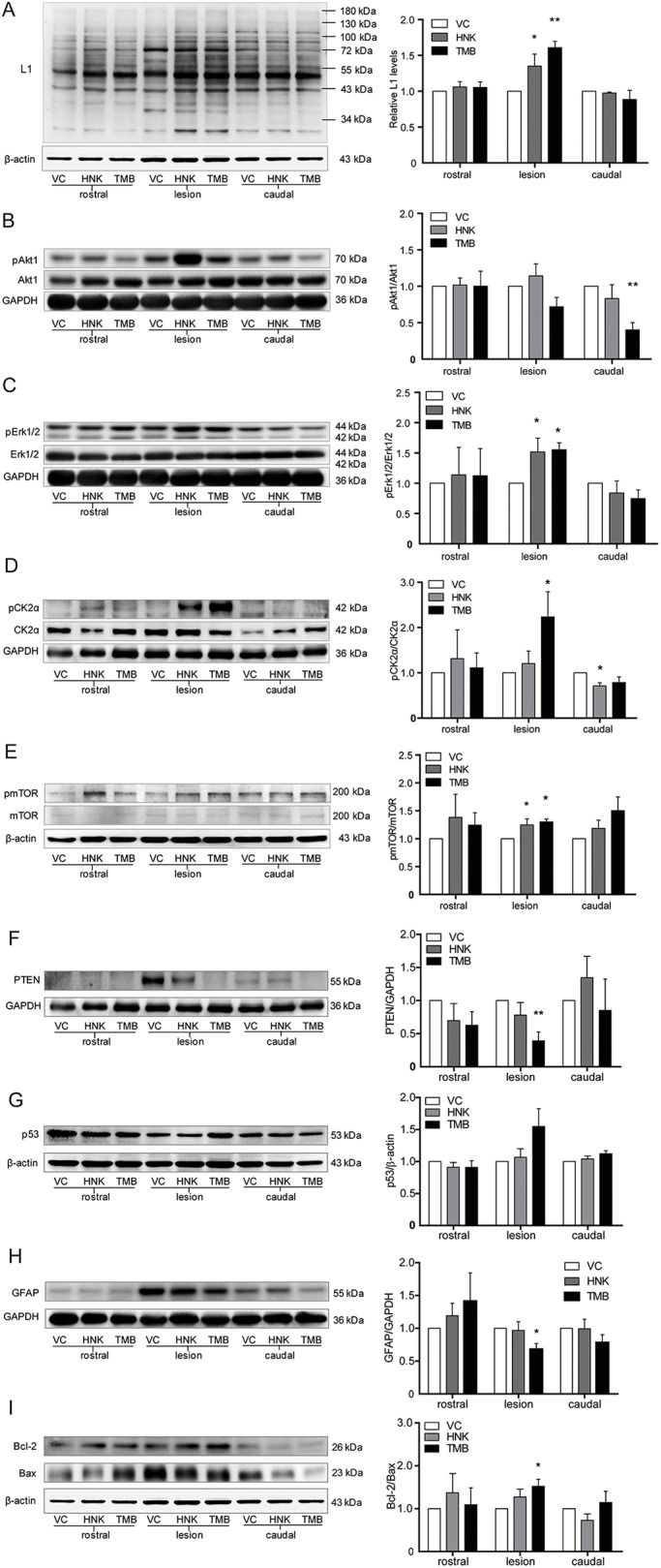
**Activation of L1-mediated intracellular signaling cascades at 6 weeks after SCI in mice treated with trimebutine or honokiol.** Western blot analysis of L1 (A), phosphorylation levels of Akt1 (B), Erk1/2 (C), CK2α (D) and mTOR (E), protein levels of PTEN (F), p53 (G) and GFAP (H), and Bcl-2/Bax ratio (I) of mice treated with vehicle control (VC), trimebutine (TMB) or honokiol (HNK) at 5 mm rostral to the lesion center, the lesion center, and 5 mm caudal to the lesion center. *P<0.05, **P<0.01 versus the vehicle control; one-way ANOVA with Tukey's post hoc test; *n*=4 mice/group.

Because trimebutine-treated, spinal cord-injured mice showed better locomotor recovery (Fig. 1), we determined whether trimebutine exerted its effects through L1-dependent cell signaling cascades by western blot analysis. The spinal cord was divided into three sections: rostral, lesion and caudal, and each section was 5 mm in length. In the lesion center, compared with vehicle control, trimebutine, but not honokiol enhanced the protein levels of all L1 immunoreactive bands (*P*=0.001 and *P*=0.047, respectively, versus vehicle control), and increased proteolytic L1 fragments with molecular weights of approximately 70, 50, 43 and 26 kDa (Fig. 6A). The compounds had no apparent effects on Akt1 phosphorylation levels in the lesion site (Fig. 6B). Erk1/2 phosphorylation levels were increased in both compound-treated groups in the central spinal cord segment containing the lesion (*P*=0.038 and *P*=0.048 for trimebutine and honokiol, respectively, versus vehicle control; Fig. 6C). In the lesion center, trimebutine increased both CK2α and mTOR phosphorylation levels (*P*=0.027 and *P*=0.011, respectively, versus vehicle control; Fig. 6D,E). Honokiol also increased the ratio of pmTOR/mTOR (*P*=0.03 versus vehicle control). PTEN and GFAP levels were decreased (*P*=0.007 and *P*=0.027, respectively, versus vehicle control) in the trimebutine group (Fig. 6F,H). Levels of p53 (Trp53) had no apparent alterations in the trimebutine and honokiol groups (Fig. 6G), but the ratio of Bcl-2/Bax was increased in trimebutine group. No obvious change was observed in the rostral and caudal sections, except that Akt1 and CK2α phosphorylation levels were decreased in the caudal sections (*P*=0.008 for trimebutine and *P*=0.027 for honokiol, respectively, versus vehicle control).

### Trimebutine and honokiol trigger L1-mediated intracellular signaling cascades in cultured cerebellar granule cells

**Fig. 7.**
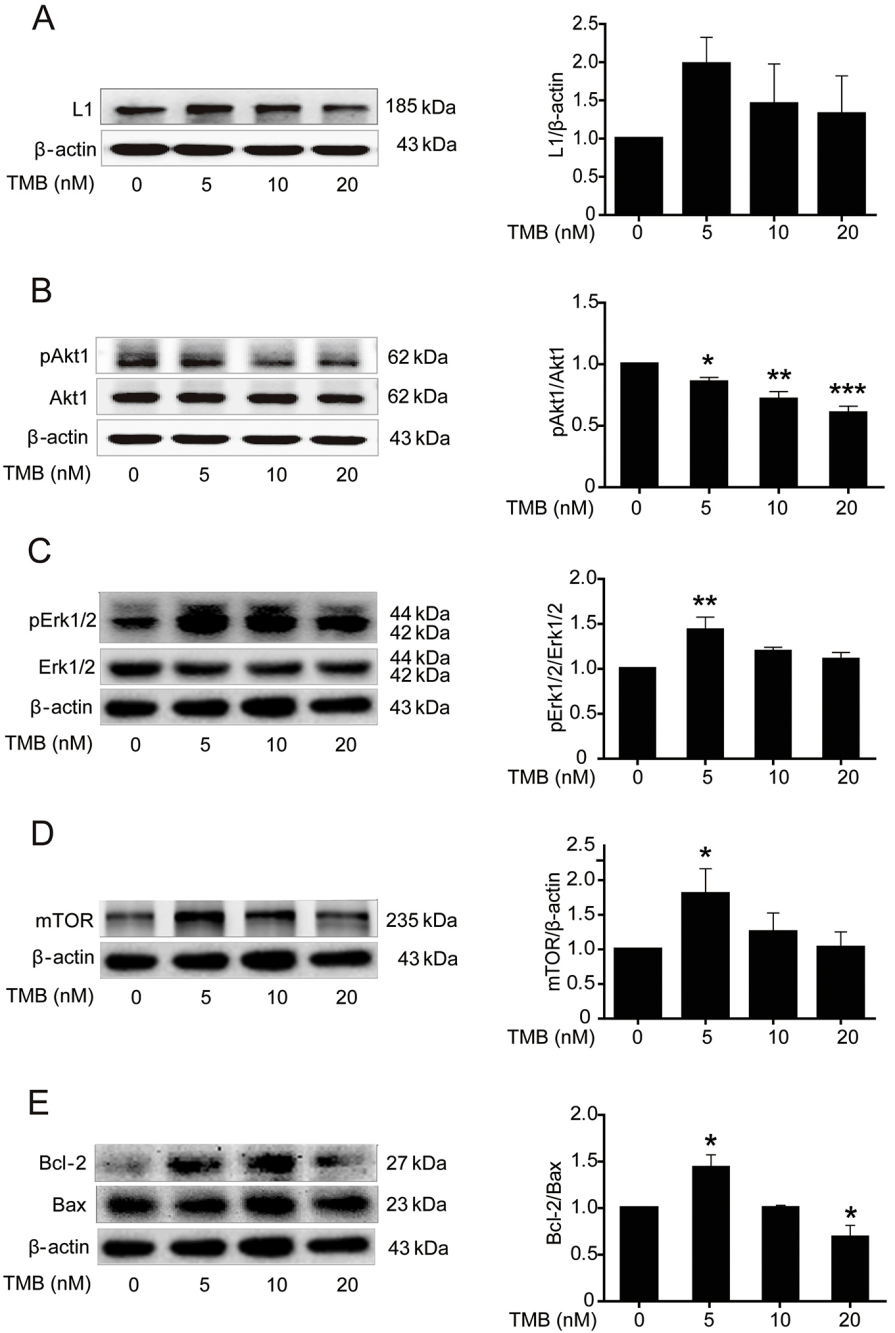
**Activation of L1-mediated intracellular signaling cascades in cultured mouse cerebellar granule cells treated with trimebutine.** (A-E) Protein levels of L1 (A), phosphorylation levels of Akt1 (B) and Erk1/2 (C), protein levels of mTOR (D), and the ratio of Bcl-2/Bax levels (E) in cultured mouse cerebellar granule cells treated with trimebutine (TMB) at concentrations of 0, 5, 10 and 20 nM. *P<0.05, **P<0.01, ***P<0.001 versus the vehicle control; one-way ANOVA with Tukey's post hoc test; three independent experiments. Blots in C-E are from the same gel and therefore have the same β-actin loading controls.

**Fig. 8.**
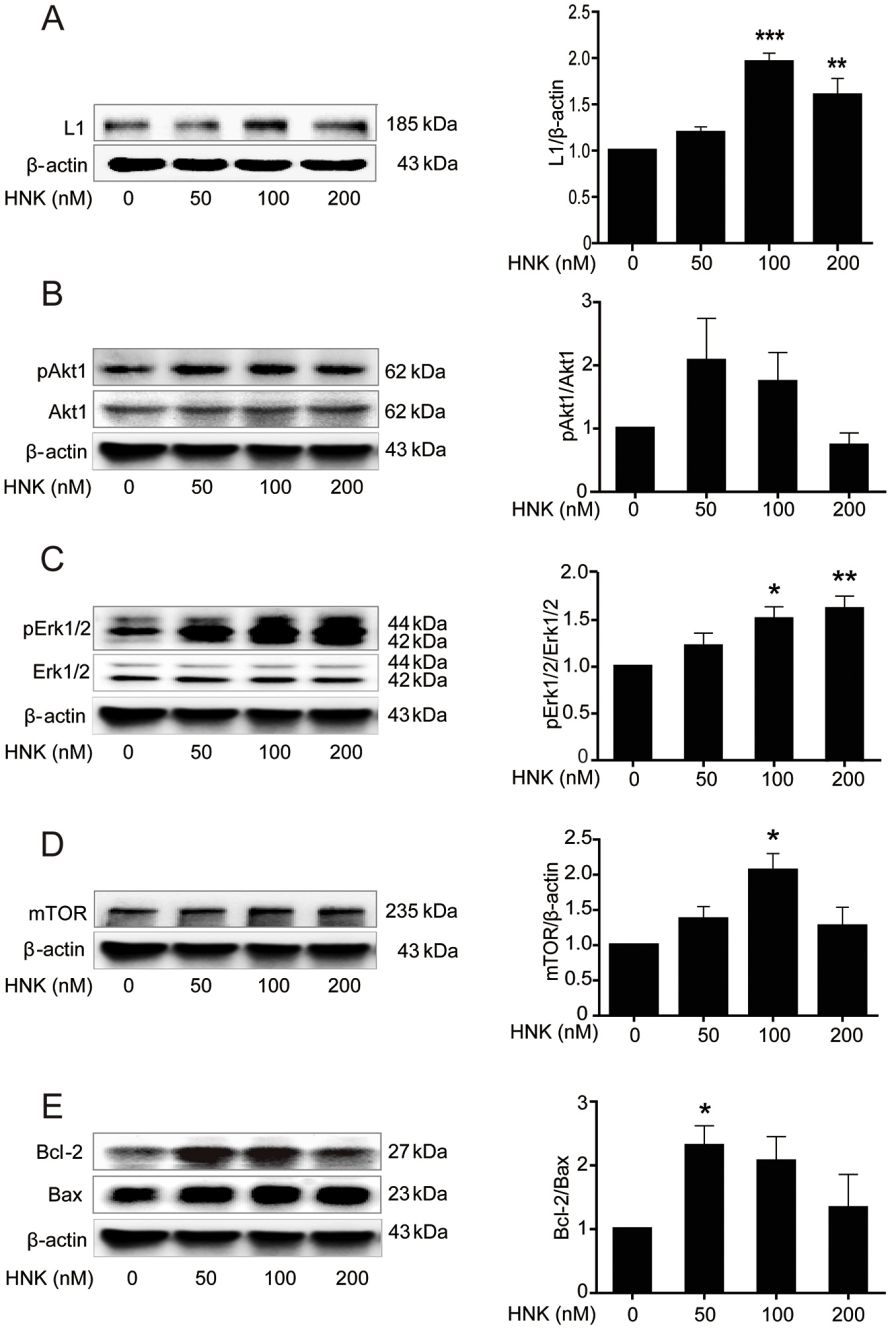
**Activation of L1-mediated intracellular signaling cascades in cultured mouse cerebellar granule cells treated with honokiol.** (A-E) L1 levels (A), phosphorylation levels of Akt1 (B) and Erk1/2 (C), protein levels of mTOR (D), and the ratio of Bcl-2/Bax levels (E) in cultured mouse cerebellar granule cells treated with honokiol (HNK) at concentrations of 0, 50, 100 and 200 nM. *P<0.05, **P<0.01, ***P<0.001 versus the vehicle control; one-way ANOVA with Tukey's post hoc test; three independent experiments. Blots in B-E are from the same gel and therefore have the same β-actin loading controls.

As the spinal cord represents a complicated tissue environment, we evaluated the effects of the compounds on L1-mediated signaling cascades in well-established cultures of early postnatal mouse cerebellar neurons by measuring the protein levels of L1 and mTOR, phosphorylation levels of Akt1 and Erk1/2, and the Bcl-2/Bax ratio in cultures of cerebellar granule cells. Keeping in mind that trimebutine and honokiol can be toxic to these cells at concentrations in the micromolar range (data not shown), trimebutine was applied at concentrations of 0, 5, 10 or 20 nM (Fig. 7), whereas honokiol treatment was with concentrations of 0, 50, 100 or 200 nM (Fig. 8). Western blot analysis in cells treated with trimebutine revealed that the levels of mTOR (Fig. 7D), phospho-Erk1/2 (Fig. 7C) and the ratio of Bcl-2/Bax (Fig. 7E) were increased, with peak changes observed with 5 nM trimebutine (*P*=0.045, *P*=0.003 and *P*=0.019, respectively, versus vehicle control). It is worth noting that the ratio of Bcl-2/Bax decreased sharply at 20 nM (*P*=0.015, versus vehicle control; Fig. 7E). Phosphorylation levels of Akt1 were dose-dependently and significantly reduced at 5, 10 and 20 nM (*P*=0.047, *P*=0.001 and *P*<0.00001, respectively, versus vehicle control; Fig. 7B). In cerebellar granule cells treated with honokiol, L1 levels increased in a dose-dependent manner, and peaked at 100 nM honokiol (*P*<0.00001 and *P*=0.004 at 100 nM and 200 nM, respectively, versus vehicle control; Fig. 8A). Similar to L1, mTOR levels peaked at 100 nM (*P*=0.02 versus vehicle control; Fig. 8D). No significant change in Akt1 phosphorylation was observed (Fig. 8B). Phosphorylation levels of Erk1/2 increased with time, and peaked at 200 nM (*P*=0.01 and *P*=0.003 for 100 nM and 200 nM, respectively, versus vehicle control; Fig. 8C). The increased Bcl-2/Bax ratio peaked at 50 nM (*P*=0.034 versus vehicle control; Fig. 8E). These observations indicate that trimebutine and honokiol can trigger L1-mediated intracellular signaling cascades in cultures of cerebellar granule cells.

## DISCUSSION

Tissue repair in the injured spinal cord faces obstacles. To complement already existing strategies aimed at reduction of inhibitory cues, our investigations pursued a more beneficial intervention that might overcome this inhibition for functional recovery. Adhesion molecule L1 is one of the beneficial molecules that fulfills the aims of promotion of neuronal survival, neuronal migration, neurite outgrowth and myelination (Chen et al., 2005, 2007). Eight small molecule L1 agonists have been identified, from a phage display peptide library, that mimic L1 functions (Kataria et al., 2016). Two of these compounds (duloxetine and piceid) not only show beneficial effects on neurons *in vitro*, but also *in vivo* after SCI. With the hope of finding even better small compounds with L1 agonistic functions, we chose another two from the available set of compounds: trimebutine and honokiol, previously shown to be effective *in vitro* (Kataria et al., 2016).

In the present study, trimebutine was more effective in enhancing recovery of hindlimb locomotor activity than was honokiol. However, as measured by GFAP expression, both of these molecules reduced the size of the glial scar to a similar extent compared with the vehicle control. Trimebutine and honokiol also both enhanced axonal regrowth/sprouting as measured by βIII-tubulin and NF200 expression. Both trimebutine and honokiol reduce immunoreactivity for GFAP surrounding the lesion site to the same extent. Hence, we suggest that the attenuated astroglial response to injury is not the underlying cause for the improved locomotor recovery seen with trimebutine. However, elevated βIII-tubulin and NF200 levels in the trimebutine group, compared with the vehicle control and the honokiol group, suggest that trimebutine promotes recovery by enhancing the regeneration of degenerating or regrowing/sprouting axons and neuronal survival (Fournier and McKerracher, 1995; Moskowitz and Oblinger, 1995). βIII-tubulin and NF200, which were not assessed in the study on duloxetine and piceid (Kataria et al., 2016), appear to be relevant parameters for the functional outcomes after injury. Overall, it appears that trimebutine leads to a better outcome after injury in comparison not only to honokiol, but also to duloxetine and piceid, although precise comparisons are difficult because of potential differences in mouse lines and animal facilities, which cannot be excluded between the two studies (Kataria et al., 2016).

The beneficial effects of L1 on long-term functional recovery after SCI have been well demonstrated (Jakovcevski et al., 2013; Kataria et al., 2016; Roonprapunt et al., 2003). L1 expression is upregulated and promotes regenerative axon regrowth/sprouting and improves behavioral outcomes after CNS injury (Schäfer and Frotscher, 2012). We observed full-length L1 and several fragments of L1 of 90, 50 and 30 kDa to be increased in the lesion site in both the trimebutine and honokiol groups compared with vehicle control. Fragments are important for neurite outgrowth, neuronal migration, survival and myelination, as demonstrated *in vitro* (Lutz et al., 2016, 2014). L1 fragments are generated by proteases, such as the matrix metalloproteinase ADAM10 and myelin basic protein (Lutz et al., 2014; Maretzky et al., 2005). The increased levels of L1 fragments in both groups also cannot be the cause for better locomotor recovery of the trimebutine versus honokiol groups.

The MAP kinase signaling pathway is required for L1-dependent neurite outgrowth (Schmid et al., 2000). For neuronal process extension, Erk plays an important role in signaling in the growth cone and nucleus. In the present study, both trimebutine and honokiol activated Erk in the lesion site, but not caudal or rostral to the lesion site. CK2 mediates both normal endocytic trafficking of L1 and L1-stimulated axon growth (Nakata and Kamiguchi, 2007), and L1-triggered CK2α activation downregulates PTEN and p53 (Wang and Schachner, 2015). PTEN suppression enhances axonal growth and functional recovery in the CNS after injury (Ohtake et al., 2015). In our study, the level of phosphorylated CK2α is increased and PTEN protein is reduced in the lesion site of the trimebutine group, possibly pointing to these molecules as one of the mechanisms underlying recovery. Interestingly, levels of pAkt1 and pCK2α are reduced caudal to the injury site, which indicates different mechanisms regarding cell death and proliferation of astrocytes and/or microglia/macrophages caudal to the lesion site versus the lesion center. Furthermore, expression of mTOR is negatively regulated by PTEN, and mTOR is expressed over a potentially crucial time scale after SCI (Kanno et al., 2012). Acutely after SCI, mTOR inhibition promotes autophagy and reduces cell death (Sekiguchi et al., 2012). In the chronic phase after SCI, activation of the mTOR pathway is sufficient to promote regenerative growth of axons (Lu et al., 2012; Sekiguchi et al., 2012). Here, we found elevated mTOR phosphorylation levels in the trimebutine group in the chronic phase as late as 6 weeks after SCI. Apoptosis is regulated by a signal transduction pathway in which Bcl-2 and Bax play key roles. Bax is involved in the induction of apoptosis and Bcl-2 has an anti-apoptotic effect. Our results show an upregulated ratio of Bcl-2/Bax in the lesion site of the trimebutine group, indicative of a distinct effect on apoptosis. Furthermore, AKT signaling is crucial in the process of resisting apoptosis and promoting the survival of neurons. Akt activation reaches a peak level 1 day after SCI, thereafter decreasing gradually (Zhang et al., 2015). No apparent change in Akt1 activation was observed in the lesion site at 6 weeks after administration of trimebutine compared with vehicle control, suggesting that Akt1 does not play the key role in trimebutine-induced repair in the chronic injury period.

The spinal cord represents a very complicated tissue environment, with protein levels changing in many cell types. To elucidate the changes of L1-mediated cell signaling pathways in neurons, we conducted *in vitro* studies with a defined cell type, under the plausible assumption that signal transduction mechanisms may be similar in different types of neurons. Motoneurons are extremely difficult to culture in sufficient amounts for western blot analysis. Therefore, the well-established culture model of mouse cerebellar granule cells was used to correlate molecular and cellular mechanisms on neuronal development and function, including survival/apoptosis and neurodegeneration/neuroprotection (Contestabile, 2002). Many fundamental insights into the processes of neuronal apoptosis, migration and differentiation in the mammalian CNS have come from investigating cerebellar granule cells (Mao et al., 1999; Selvakumar and Kilpatrick, 2013; Shalizi et al., 2003)*.* Proteins such as Akt1 and Erk may exert different functions in neurons and glial cells. For instance, Akt signaling is crucial in resisting apoptosis and promoting the survival of neurons. Multiple studies of neuronal apoptosis have frequently relied on cultures of granule cells, exploiting their responses to activity and growth factor deprivation as well as oxidative stress (Li et al., 2001). Apoptosis is regulated by a signal transduction pathway in which Bcl-2 and Bax play key roles. In addition, the cerebellum plays important roles in the control and coordination of movements and motor functions. Thus, evaluation of the effect of L1 mimetic treatment on cerebellar granule cell function and signaling will improve our understanding of the mechanisms that underlie recovery of motor functions *in vivo*. The protein levels of L1-activated MAPK and PI3 kinase pathways were examined. L1-mediated intracellular pathways were activated after trimebutine treatment at 5 nM and honokiol treatment at 50 or 100 nM, suggesting that cells are more sensitive to trimebutine treatment compared with honokiol. Future studies will need to adjust the concentrations of trimebutine and honokiol to reach functionally optimal levels after SCI. It is noteworthy in this context that trimebutine leads to more cell toxicity than honokiol, as shown by the Bcl-2/Bax ratio for the compounds acting on cerebellar granule cells. Trimebutine at 5 nM increased the Bcl-2/Bax ratio, whereas at 20 nM it decreased the Bcl-2/Bax ratio, compared with vehicle control, indicating that a low concentration of trimebutine reduces apoptosis, but induces apoptosis at a higher concentration. This may partly explain why the levels of L1, pErk/Erk and mTOR reach their peak levels at 5 nM of trimebutine, gradually decreasing at higher trimebutine concentrations.

Trimebutine exhibits anti-muscarinic and weak μ, κ and δ opioid agonist effects (Delvaux and Wingate, 1997; Kaneto et al., 1990). The muscarinic acetylcholine receptor plays a crucial role in afferent bladder activation that enhances detrusor overactivity in spinal cord-injured rats (Matsumoto et al., 2012), but this effect does not influence regeneration after spinal cord injury, so it is unlikely to be relevant for the observations in the present study. The trimebutine metabolite nor-trimebutine was shown to decrease Fos activation in the spinal cord and the sacral parasympathetic nucleus after chronic inflammation and this effect was suggested to underlie the decrease of abdominal contractions and abdominal pain after trimebutine treatment (Sinniger et al., 2005). However, it was also suggested that activation and upregulation of Fos might be necessary for regeneration after SCI (Sabin et al., 2015). Therefore, it is unclear whether up- or downregulation of Fos is beneficial for regeneration in the mammalian spinal cord. As Kataria et al. (2016) showed that honokiol and trimebutine are unable to stimulate neurite outgrowth and neuronal survival of L1-deficient neurons, these drugs are likely to stimulate these cellular processes via L1. Although we cannot exclude the possibility that non-L1-related mechanisms contribute to the effect of trimebutine in the SCI model, our findings show that L1-mediated signaling pathways do play a relevant role in trimebutine-induced regeneration after SCI. Of note, honokiol improves functional recovery after traumatic brain injury in rats, but this effect has not been compared with trimebutine treatment (Wang et al., 2014). We thus suggest that trimebutine might not only be beneficial for functional recovery in SCI, but also in other central nervous injury models involving L1-dependent functions, and could be a promising therapeutic intervention for injured humans.

## MATERIALS AND METHODS

### Animals and reagents

Specific pathogen-free female mice (wild type C57BL/6, 3 months old) were purchased from the Guangdong Medical Animal Centre (People's Republic of China). All experiments and protocols were approved by the Ethics Committee of Shantou University Medical College, and all experiments were conducted in accordance with authorities of the Guangdong province. Trimebutine (sc-204928, Santa Cruz) and honokiol (4590, Tocris Bioscience) were dissolved in dimethyl sulfoxide (DMSO; 196055, MP Biomedicals, France) and then diluted in 0.9% saline solution. DMSO needed to be used in the experiments, because the compounds are not soluble in purely physiological vehicles. Trimebutine and honokiol were administered via the tail vein at a dose of 1 mg per kg body weight (total volume 50 μl), respectively. All efforts were made to minimize the suffering of animals and to reduce the number of animals used in the experiments.

### Spinal cord injury

Compression of the spinal cord injury was performed as described (Pan et al., 2014). Briefly, mice were anesthetized by intraperitoneal injections of ketamine and xylazine (100 mg Ketanest, Gutian Pharmaceutical, Fujian, China, and 5 mg Rompun, Sigma, per kilogram body weight). Laminectomy was carried out at the T7-T9 levels with mouse laminectomy forceps (Fine Science Tools, Heidelberg, Germany). The spinal cord was manually exposed, and then maximally compressed with forceps (100%) according to the operational definition as described (Curtis et al., 1993) for 5 s to cause a robust and reproducible lesion. After the surgery, mice were injected intraperitoneally with 200 μl 0.9% saline solution as supplementary body liquid. Mice were divided randomly into three groups (seven mice per group). The vehicle group received 0.1% DMSO diluted in 0.9% saline solution, and the other two groups received trimebutine and honokiol (1 mg/kg) through the tail vein at 15 min (the shortest time practically possible after injury) after SCI and thereafter daily from 2 to 7 days. During the post-operative period, the bladders of the animals were emptied twice daily or as needed.

### Analysis of motor function

The Basso Mouse Scale (BMS) score was used to measure functional recovery of ground locomotion at the individual animal level (Basso et al., 2006). Furthermore, single-frame motion analysis was applied as a more precise assessment of locomotion to determine the foot-stepping angle and rump-height index, using beam walking, and to measure the extension/flexion ratio, using pencil catching (Apostolova et al., 2006). The left and right extremity view of each mouse during two consecutive walking trials were recorded, prior to SCI and 1, 2, 3, 4, 5 and 6 weeks after injury with a high-speed video camera, and analyzed with the affiliated analysis software (SIMI Motion, SIMI Reality Motion Systems, Unterschleissheim, Germany). Values for the left and right limbs were averaged. We calculated and analyzed relative estimates of functional recovery except the absolute values of the functional parameters mentioned above. Recovery index (RI) was used as an estimate of functional recovery at the individual animal level as described (Pan et al., 2014). Overall RI was calculated, on an individual animal basis, as means of RI for BMS score, foot-stepping angle and rump-height index. RI is considered as a ‘clinical score’ for individual mice, similar to the BMS score, which is based on assessment of different aspects of locomotion.

### Tissue fixation and sectioning

Six weeks after SCI, mice were deeply anesthetized with isoflurane, and then perfused with 50 ml 0.9% saline solution followed by 50 ml 4% formaldehyde (dissolved from paraformaldehyde in 0.1 M PBS, pH 7.4) through the left cardiac ventricle. The vertebral column was dissected out and post-fixed in 4% formaldehyde solution for 1 week. The spinal cord was peeled out, immersed in 20% sucrose solution in PBS for 48 h at room temperature (RT) and then embedded in Tissue-Tek (4583, Sakura Finetek, Torrance, USA). Serial longitudinal coronal sections of the mouse spinal cord at a thickness of 8 μm were obtained using a cryostat (CM1860, Leica, Nussloch, Germany), and sampling of sections was always performed in a standard sequence so that sections were 80 µm apart and placed together on one glass slide for standard comparison.

### H&E staining

H&E staining was performed as described (Schreiber et al., 2013). After soaking in PBS for 5 min, spinal cord sections were stained with Hematoxylin (ZLI-9609, ZSGB-Bio, Beijing, China) for 10 s, washed with water for 5 min, placed in a humidified box for 1 h at RT, and then stained with Eosin (ZLI-9613, ZSGB-Bio, Beijing, China) for 5 min. After washing in water for 5 min, sections were mounted using the neutral balsam mounting medium (ZLI-9516, ZSGB-Bio, Beijing, China). All sections were examined under light digital microscopy (DM-117M, Jiangnan, Jiangsu, China).

### Immunohistochemistry (IHC)

Antigen retrieval for spinal cord sections was carried out in citrate buffer (0.01 M, pH 6.0) for 40 min at 99°C in a water bath. Endogenous peroxidase activity was inhibited by incubating in 3% H_2_O_2_ solution for 10 min at RT. Non-specific binding sites were blocked using normal goat serum (AR0009, Boster) for 30 min at RT. Sections were thereafter incubated with rabbit polyclonal anti-glial fibrillary acidic protein (GFAP, 1:500, BA0056, Boster), mouse monoclonal anti-βIII-tubulin (1:200, sc-80016, Santa Cruz), mouse monoclonal anti-cell adhesion molecule L1 (1:200, MAB777, R&D Systems), rabbit polyclonal anti-mTOR (1:200, sc-8319, Santa Cruz), mouse monoclonal anti-p53 (1:1000, sc-98, Santa Cruz), or mouse monoclonal anti-PTEN (1:100,sc-7974, Santa Cruz) antibodies overnight at 4°C. After washing in PBS three times for 5 min each at RT, sections were visualized with a DAB Detection Kit (PV-9000-D, ZSGB-Bio, Beijing, China) and then stained in the dark using a 3-amino-9-ethylcarbazole (AEC) kit (0037, Maixin Biotech, Fuzhou, China) according to the manufacturer's protocols. Stained sections were mounted in water-soluble mounting medium (AR1018, Boster). IHC images were taken under a light digital microscope (DM-117M, Jiangnan, Jiangsu, China) 24 h after the slides were mounted.

### Immunofluorescence staining

Antigen retrieval for spinal cord tissue sections was conducted using 10 mM citrate buffer (pH 6.0) at 99°C for 40 min. Non-specific protein binding sites were blocked by incubation with 10% normal donkey serum diluted in PBS at RT for 1 h. The sections were incubated at 4°C overnight with GFAP and βIII-tubulin, L1 or rabbit anti-pCK2α (1:100, phospho-Thr360/Ser362, 11572, SAB Biotech, MD, USA), GFAP or mouse anti-neurofilament 200 (NF200, BM0100, 1:1000, Boster) antibodies. After washing with PBS three times for 5 min each, the samples were incubated with a donkey anti-mouse secondary antibody conjugated to Dylight 488 (1:500, 715-545-150, Jackson ImmunoResearch Laboratories, West Grove, PA, USA) and a donkey anti-rabbit secondary antibody conjugated to Dylight 594 (1:1000, 711-585-152, Jackson ImmunoResearch Laboratories) at RT in the dark for 90 min. The samples were finally mounted using ProLong Gold Antifade reagent with DAPI (P36935, Life Technologies). Double-immunofluorescence images were acquired with an Olympus laser confocal system (FV-1000, Olympus, Japan). DAPI was excited at 405 nm. Dylight 488 and Dylight 594 were excited at 488 nm and 594 nm, respectively, in a multi-track configuration.

### Quantification of immunohistology

The contusion site was defined as the lesion center as it showed a different optical density compared with the adjacent tissue, as indicated by H&E staining and immunohistochemical analysis. The rostral and caudal regions were taken in cross-sections 1 mm away from the margin of the lesion center. Quantification of the lesion site region (shown by H&E staining), positive immunoreactive density (shown by immunohistochemical staining) and immunofluorescence intensity (shown by immunofluorescence staining) of detected molecules at the contusion site or 1 mm rostral or 1 mm caudal to the lesion center were analyzed using Image-Pro Plus 6.0 (Media Cybernetics). Lesion scar size was estimated by measuring integrated optical density in the area of interest from H&E-stained sections. For each animal, three equidistant (80 μm apart) longitudinal sections were stained for analysis of the target molecule.

### Primary culture of cerebellar granule cells

Cerebella were obtained from C57BL/6 mice at postnatal day 7 and chopped after removal of the vessels and meninges (Loers et al., 2014b). Tissues were dissected, kept in cold DMEM/F12 (HyClone, Thermo Fisher Scientific) in a 15 ml conical tube on ice, and then digested with Hank's balanced salt solution (HBSS, H1045-500, Solarbio, Beijing, China) and trypsin (0.25%) at 37°C in a humidified 5% CO_2_ atmosphere for 30 min. Digestion was terminated by addition of 1 ml 10% fetal bovine serum (HB0205, Sijiqing Biotech, Hangzhou, China) in DMEM/12. The mixture was triturated and centrifuged at 1200 ***g*** at 4°C for 5 min. Supernatant was discarded, and the single cells were inoculated onto 48-wells pre-coated with 100 µg/ml poly-D-lysine in a volume of 300 µl containing 1×10^5^ cells, cells were cultured in DMEM/F-12medium supplemented with 10% fetal bovine serum and 1% penicillin/streptomycin (Solarbio Biotech Corp) at 37°C in a 5% CO_2_ incubator for 6 h to enable cell adhesion to the plates. Next, the medium was aspirated and replaced with Neurobasal-A (Life Technologies) culture medium supplemented with 2% B-27 (Life Technologies) and 1% penicillin/streptomycin. To investigate the molecular mechanism of L1 signaling *in vitro* at the protein level, the cultured cells were treated with 0, 5, 10 or 20 nM trimebutine, or with 0, 50, 100 or 200 nM honokiol. Whole cell lysates were collected after culturing for 48 h.

### Western blot analysis

All spinal cord tissue lysates and cell lysates were combined with a 20% sample loading buffer and heated for 15 min at 95°C. Protein samples were resolved using 10% sodium dodecyl sulfate-polyacrylamide gel electrophoresis and then electroblotted onto polyvinylidenedifluoride membranes. Non-specific protein binding sites were blocked with 5% nonfat milk or bovine serum albumin diluted in Tris-HCl saline buffer supplemented with 0.1% Tween-20 (TBST, pH 7.4) for 1 h. Membranes were incubated with antibodies specific for mouse monoclonal anti-L1 (1:1000, MAB777, R&D Systems), rabbit polyclonal anti-p-mTOR (1:1000, sc-101738, Santa Cruz), rabbit polyclonal anti-mTOR (1:1000, sc-8319, Santa Cruz), rabbit polyclonalanti-pCK2α (1:1000, phospho-Thr360/Ser362, 11572, SAB Biotech), mouse monoclonal anti-CK2α (1:200, sc-365763, Santa Cruz), rabbit polyclonal anti-GFAP (1:2000, BA0056, Boster), mouse monoclonal anti-pAkt1 antibody (1:1000, sc-81433, Santa Cruz), mouse monoclonal anti-Akt1 (1:1000, sc-55523, Santa Cruz), mouse monoclonal anti-pErk1/2 (1:1000, sc-7383, Santa Cruz), mouse monoclonal anti-Erk1/2 (1:1000, sc-135900, Santa Cruz), rabbit polyclonal anti-Bax (1:1000, sc-526, Santa Cruz), mouse monoclonal anti-Bcl-2 (1:1000, sc-7382, Santa Cruz), mouse monoclonal anti-p53 (1:1000, sc-98, Santa Cruz) and mouse monoclonal anti-PTEN (1:200, sc-7974, Santa Cruz) with gentle agitation overnight at 4°C. After washing the membrane with 0.1% TBST 3 times for 5 min each at RT, membranes were further incubated with horseradish peroxidase (HRP)-conjugated goat anti-mouse secondary antibody (1:1000, BA1051, Boster) or anti-rabbit secondary antibody (1:1000, BA1055, Boster) for 1 h, followed by washing three times for 5 min each with 0.1% TBST. Immunoreactive bands were visualized using an enhanced chemiluminescence kit (WBKLS0500, Millipore). The signal intensity was quantified using ImageJ software.

### Statistical analysis

Statistical analysis was performed using SPSS 17.0 software. All numerical data are presented as mean±s.e.m. unless otherwise indicated. Differences among variables were assessed by one-way ANOVA with Tukey's post hoc test. Values of *P*<0.05 were considered statistically significant. BMS scoring and histological analyses were performed with the operator blind to treatment.
